# Effects of Ammonium Perchlorate on Thyroid Function in Developing Fathead Minnows, *Pimephales promelas*

**DOI:** 10.1289/ehp.7333

**Published:** 2005-01-10

**Authors:** Helen M. Crane, Daniel B. Pickford, Thomas H. Hutchinson, J. Anne Brown

**Affiliations:** ^1^School of Biological Sciences, Hatherly Laboratories, University of Exeter, Exeter, United Kingdom; ^2^AstraZeneca, Global Safety, Health and Environment, Brixham Environmental Laboratory, Brixham, Devon, United Kingdom

**Keywords:** development, endocrine disruption, fathead minnow, perchlorate, thyroid, thyroxine, triiodothyronine

## Abstract

Perchlorate is a known environmental contaminant, largely due to widespread military use as a propellant. Perchlorate acts pharmacologically as a competitive inhibitor of thyroidal iodide uptake in mammals, but the impacts of perchlorate contamination in aquatic ecosystems and, in particular, the effects on fish are unclear. Our studies aimed to investigate the effects of concentrations of ammonium perchlorate that can occur in the environment (1, 10, and 100 mg/L) on the development of fathead minnows, *Pimephales promelas*. For these studies, exposures started with embryos of < 24-hr postfertilization and were terminated after 28 days. Serial sectioning of thyroid follicles showed thyroid hyperplasia with increased follicular epithelial cell height and reduced colloid in all groups of fish that had been exposed to perchlorate for 28 days, compared with control fish. Whole-body thyroxine (T_4_) content (a measure of total circulating T_4_) in fish exposed to 100 mg/L perchlorate was elevated compared with the T_4_ content of control fish, but 3,5,3′-triiodothyronine (T_3_) content was not significantly affected in any exposure group. Despite the apparent regulation of T_3_, after 28 days of exposure to ammonium perchlorate, fish exposed to the two higher levels (10 and 100 mg/L) were developmentally retarded, with a lack of scales and poor pigmentation, and significantly lower wet weight and standard length than were control fish. Our study indicates that environmental levels of ammonium perchlorate affect thyroid function in fish and that in the early life stages these effects may be associated with developmental retardation.

In recent years there has been increasing concern about the presence of perchlorate in ground and surface waters and the percolation of perchlorate into drinking waters [Urbansky 1998; U.S. Environmental Protection Agency (EPA) 2002]. Ground and surface waters in several U.S. states have shown measurable concentrations of perchlorate at 8 μg/L to 3.7 g/L (Urbansky 1998). The major source of ground and surface water contamination is ammonium perchlorate, the primary ingredient of the solid propellant in rockets and missiles (Logan 2001; U.S. EPA 2002). Perchlorate salts are also used in smaller amounts as components of air bag inflators, road flares, and fireworks; in electroplating and in tanning and finishing leathers; and as mordants for fabrics and in producing paints and enamels (Logan 2001; U.S. EPA 2002. Discharge from rocket fuel manufacturing plants, demilitarization of weapons, and the washing out and refueling of rockets are responsible for most of the ammonium perchlorate released into the environment (Urbansky 1998; U.S. EPA 2002). Indeed, at the Longhorn Army Ammunition Plant in Texas (USA), perchlorate has been measured at 30–31 mg/L in a water treatment holding pond (Smith et al. 2001).

Perchlorate has several chemical properties that make environmental contamination difficult to resolve and decontamination difficult to achieve (Logan 2001). The perchlorate anion is persistent because of its tetrahedral structure (Wolff 1998). Perchlorate salts completely ionize in solution, and the perchlorate anion is highly mobile (Logan 2001). As a result of these properties, groundwater contamination inevitably presents a risk to drinking water quality, and perchlorate has been detected in many drinking water supplies. In Nevada, 4–24 μg/L was detected in drinking water (Xiao et al. 2001), and in California a number of drinking water wells showed peaks of 4–820 μg/L (California Department of Health Services 2004). As a result, the U.S. EPA has estimated that perchlorate affects the quality of drinking water for 15 million people in the United States (Logan 2001).

Based on U.S. EPA guidance, and assessment of toxicity data, several U.S. states have set advisory levels for perchlorate in drinking water that vary between 1 and 18 μg/L. The most recent reappraisal in California set a public heath goal for drinking water (maximum contaminant level) of 6 μg/L (Office of Environmental Health Hazard Assessment 2004).

There is a long history of clinical use of perchlorate as a pharmacologic inhibitor of thyroid hormone synthesis (Hobson 1961; Wolff 1998). Thyroid gland follicles trap iodide required for the iodination of tyrosine molecules. The resulting iodothyronines are then reversibly combined with the storage protein, thyroglobulin, within the lumen of each of the thyroid follicles (Leatherland 1988, 1993). Perchlorate competitively inhibits iodide uptake by the sodium/iodide symporter at the basolateral membrane of the follicles (Capen 1997; Wolff 1998) and induces iodide efflux from the follicles by an as yet unexplained mechanism (Wolff 1998). These pharmacologic actions might be predicted to reduce circulating levels of thyroid hormones, and several studies in mammals given drinking water containing perchlorate at target doses of 0.01–100 mg/kg/day support this idea (Siglin et al. 2000; York et al. 2001a). However, this has not been observed in all studies (York et al. 2001b).

Despite the known action of perchlorate on iodide uptake by the mammalian thyroid gland (Capen 1997; Wolff 1998) and evidence of perchlorate occurrence in aquatic ecosystems, remarkably few studies have investigated the effects of perchlorate on thyroid function of aquatic vertebrates. In the amphibian *Xenopus laevis*, 5 μg/L perchlorate inhibited forelimb emergence during thyroid-dependent metamorphosis (Goleman et al. 2002), and at 18 μg/L significantly fewer *Xenopus* completed tail resorption. Metamorphosis of larval sea lamprey *Petromyzon marinus* (an agnathan fish) is also affected by exposure to perchlorate (Manzon and Youson 1997, 2002; Manzon et al. 2001), but only two studies have investigated the impact of perchlorate on teleost fish that form key components of freshwater ecosystems. Potassium perchlorate (500 mg/L) was found to inhibit fin formation and skin pigment differentiation in early life stages of zebrafish, *Danio rerio* (Brown 1997), and in a study of adult zebrafish a high level of ammonium perchlorate (18 mg/L) resulted in thyroid hypertrophy, hyperplasia, and colloid depletion after 8 weeks of exposure (Patino et al. 2003). However, neither of these studies determined the changes in circulating thyroid hormones or whole-body thyroid hormone content. Our studies aimed to gain an integrated picture of the impacts of environmentally relevant concentrations of ammonium perchlorate on the thyroid axis of teleost fish by measuring whole-body thyroxine (T_4_) and 3,5,3′-triiodothyronine (T_3_) content, and examination of thyroid structure, together with investigation of changes in development and growth. For our studies we employed the fat-head minnow as a model cyprinid species. Although this species has been widely used in ecotoxicologic studies, knowledge of its thyroid function and the normal levels and fluctuations of T_4_ and T_3_ has only recently been obtained (Crane 2003; Crane et al. 2004). These data have shown a surge in thyroid hormones during the early stages of development and indicate that exposure to thyroid disruptors may have greatest effect during early development. Therefore, in investigating the effects of perchlorate, we focused on the first 28 days of development from < 24 hr postfertilization through the transition to juvenile fish.

## Materials and Methods

### Semistatic exposure system.

In a 28-day semi-static study, fathead minnow newly fertilized eggs were exposed to ammonium perchlorate in triplicate at 0 (control), 1, 10, and 100 mg/L (equivalent to 0.85, 8.47, and 84.7 mg/L perchlorate anion) with 0.15, 1.53, and 15.3 mg/L NH_4_^+^ in the experimental systems. The presence of added NH_4_^+^ would have resulted in 0.5 μg/L, 5.1 μg/L, and 51 μg/L unionized ammonia in the three experimental systems, but these levels of ammonia are well below the chronic-effects threshold concentration for unionized ammonia in fathead minnow larvae and adults (270 μg/L) based on a 2-year full-life cycle assessment of survival, growth, and reproductive success (Thurston et al. 1986).

From study day 0 to day 13, groups of fertilized eggs (*n* = 60) were exposed to perchlorate in 2-L Pyrex glass beakers (working volume, 1 L). Most embryos hatched on study day 5. On posthatch day 8 (study day 13), fish were transferred to 3-L Pyrex glass beakers (working volume, 2 L). Finally, on posthatch day 15 (study day 20) fish were transferred to 12-L glass tanks (working volume, 3 L) for the remainder of the study.

### Preparation of stock concentrate and test solutions.

One day before the start of the study, ammonium perchlorate stock concentrate (50 g/L) was made up in dechlorinated local tap water (filtered to 10 μm, maintained at 25 ± 2°C). The stock was renewed at 11-day intervals. Typical water quality parameters were hardness, 44.7–49.3 mg/L; free chlorine, < 2.0 μg/L; calcium, 14.1 mg/L; sodium, 28.6 mg/L; potassium, 1.43 mg/L; aluminum, 4.73 μg/L; iron, < 3.0 μg/L; lead, < 2.0 μg/L. Test solutions (nominal concentrations of ammonium perchlorate, 0, 1, 10, and 100 mg/L) were made from the stock concentrate by dilution in dechlorinated water. Test and control solutions were renewed on study day 5 (principal hatch day) and then three times per week thereafter.

### Embryo selection, maintenance, and exposure.

On day 0, fathead minnow embryos (*n* = 240) at blastula and morula developmental stages were gathered from mating tiles in the husbandry unit at Brixham Environmental Laboratory. Healthy embryos were selected, in batches of five, under a dissection microscope and randomly assigned to incubation cups, until each contained 10 embryos. Incubation cups were hung on oscillating incubation units over the fish tanks, maintaining the embryos constantly moving up and down through the test water at 2 cycles/min. There were two egg cups per tank, each containing a total of 10 embryos (20 embryos per tank), and a total of 60 embryos per exposure concentration, in triplicate. Embryos were inspected on a daily basis, and dead ones were removed. Most of the larvae hatched on study day 5 and were transferred to new test solution.

### Maintenance of fry.

From posthatch day 0 to day 6, larvae were fed a suspension of rotifers (3,000 rotifers/mL; 2 mL three times per day on Monday–Friday and twice per day on weekends). On posthatch day 7, larvae were fed *Artemia nauplii* as well as rotifers. During posthatch days 8–15, fry were fed *Artemia* only, three times per day, and then from posthatch day 16 to the end of the study fry were fed *Artemia* twice per day and ground pellets (Ecostart 17; Biomar, Grangemouth, Scotland) once per day.

### Sampling regime.

On study day 28, fish were sacrificed between 06:00 and 10:00 hr by immersion in a lethal dose of neutrally buffered ethyl 3-amino-benzoate methane sulfonate (MS222). Each fish was measured using digital calipers to determine standard length, weighed, and then snap frozen on dry ice. From each exposure concentration, 20 fish were sampled for whole-body thyroid hormone analyses, and a further 10 fish in each exposure group were fixed by immersion in 10% phosphate-buffered formalin for histologic examination of thyroid follicles, taking fish with an even distribution between the triplicate tanks. Fish for thyroid hormone assays were held at −80°C until analysis.

### Histology.

Whole fish (*n* = 5) fixed in formalin were decalcified for 14 days in 5% formic acid in 5% formaldehyde. Fish were wax embedded and serially sectioned (6 μm) through all the thyroid follicles. Each follicle in each fish (5–13 follicles/fish) was traced through its entirety, and epithelial cell height was measured at the largest point.

### Thyroid hormone extractions.

Thyroid hormones were extracted from fathead minnow larvae based on the technique described by Greenblatt et al. (1989). Larvae were placed in Teflon tubes on ice, and 2 mL 95% ethanol containing 1 mM 6-*N*-propyl-2-thiouracil (PTU) was added. Samples were homogenized (Ultra Turax T25; Janke and Kunkel, Staufen, Germany) and sonicated for 20 sec (Vibra-Cell, 50% output; Sonics and Materials, Meryin/Satigny, Switzerland). A further 2 mL of 95% ethanol with 1 mM PTU was added, and samples were vortexed. Samples were centrifuged for 10 min (10,000*g*, 4°C), the supernatant was decanted into clean Teflon tubes, and 2 mL 95% ethanol containing PTU was added to the pellets. Tubes were vortexed vigorously and recentrifuged for 10 min (10,000*g*, 4°C). Supernatants were pooled and evaporated to dryness under nitrogen, and desiccated samples were resuspended in 0.25 mL barbital buffer containing 2.5 mg/mL anilino naphthalene sulfonic acid (to disrupt the coupling between thyroid hormones and serum proteins, including lipoproteins), 0.25 mL ethanol, and 1 mL chloroform. Tubes were vigorously vortexed and then centrifuged for 10 min (1,500*g*, 4°C), producing two phases. The top ethanolic layer was removed using a glass pipette for radioimmunoassay (RIA) of thyroid hormones. The recovery of thyroid hormones was determined by addition of radioiodinated T_4_ or T_3_ after homogenization of whole larvae (*n* = 5). The recovery of 59.5 ± 3.25% T_4_ and 63.9 ± 3.27% T_3_ was comparable with those recoveries reported for larvae of other fish species (Greenblatt et al. 1989).

### Radioimmunoassay of T_3_ and T_4_.

The RIAs used in these studies were developed and validated for use with fathead minnows (Crane 2003). For these assays, thyroid hormone standards (0.22–160 ng/mL T_4_; 0.06–10 ng/mL T_3_) were prepared in barbital buffer. Lyophilized polyclonal anti-T_3_ and anti-T_4_, raised in sheep (Diagnostics, Edinburgh, Scotland), were diluted in barbital buffer (1:7,000 for anti-T_4_, 1:10,000 for anti-T_3_). Radioiodinated T_3_ (I^125^-T_3_) and T_4_ (I^125^-T_4_; specific activities, 1,080–1,320 μCi/μg; New England Nuclear, Boston, MA, USA) were used at approximately 5,000 counts per minute (cpm) per tube for I^125^-T_3_ and 6,500 cpm per tube for I^125^-T_4_.

Extracted samples or standard solutions (30 μL) were incubated at 4°C overnight (in triplicate) with 100 μL antiserum and 100 μL radioiodinated solution, with additional “total counts” and “blank” tubes. The next morning, free and bound hormones were separated by addition of 100 μL Sac-Cel (Immunodiagnostic Systems Limited, Tyne and Wear, UK) and a solution of cellulose-coupled antibodies (anti-sheep/goat); tubes were centrifuged, and the pellet of bound radiolabeled hormone was counted (Cobra gamma counter; Packard, Boston, MA, USA). Thyroid hormone levels were estimated using RIA software (RIASMART; Packard).

**Figure 1:**
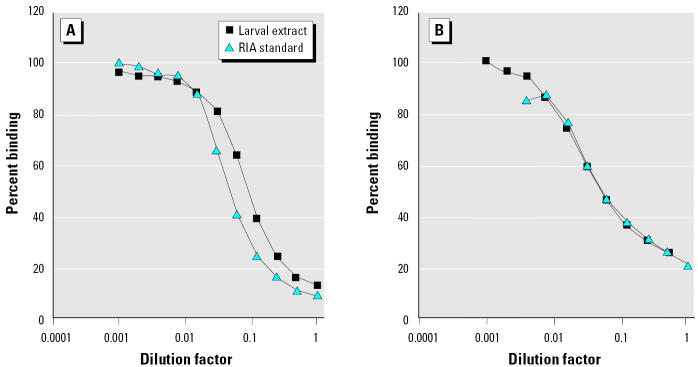
Parallelism between serially diluted larval extract and standards in the RIAs for (*A*) T_4_ and (*B*) T_3_.

The thyroid hormone RIAs were validated for the estimation of thyroid hormones extracted from larvae, by running serial dilutions of an extracted pool of larvae in both T_3_ and T_4_ RIAs. These assays showed parallelism of larval extracts with the two standard curves (Figure 1). The cross-reaction of anti-T_3_ and anti-T_4_ with thyroid hormone metabolites [reverse triiodothyronine (rT_3_), diiodothyronine, monoiodotyrosine, and tyrosine] was determined by running serial dilutions in the two thyroid hormone RIAs. Anti-T_4_ showed 1.24% cross-reaction with T_3_ and 2.38% cross-reaction with rT_3_. Anti-T_3_ showed 5% cross-reaction with T_4_, 0.02% cross-reaction with rT_3_, and 3.4% cross-reaction with diiodothyronine. Other metabolites produced no displacement in the assay and thus exhibited negligible cross-reaction. The minimum detectable level of thyroid hormones, estimated as the mean plus two standard deviations of zero standards (*n* = 18 for T_4_, *n* = 9 for T_3_) was 2.04 pg/tube for T_3_ and 8.16 pg/tube for T_4_, which equates to whole-body contents of approximately 0.35 pg/mg T_3_ and 1.41 pg/mg T_4_. All RIA measurements were acquired in a single T_3_ assay and a single T_4_ assay. Intra-assay variation for the T_3_ RIA was 9.68% (*n* = 12), and for the T_4_, RIA was 5.33% (*n* = 15).

### Statistical analysis.

All data shown are mean ± SEM. Percentage survival and percentage hatch data were arcsine square root transformed before statistical analyses with modification where *n* = 0 or 1, as detailed by the U.S. EPA (1994), and analyzed by one-way analysis of variance (ANOVA) with post hoc Tukey honestly significantly different (Tukey HSD) tests. Wet weight, length, thyroid hormone content (adjusted for extraction efficiency), and follicular cell heights were analyzed using Kruskal-Wallis one-way ANOVA on ranks with Dunn’s test for multiple comparisons.

## Results

### Hatch and survival.

All test vessels showed hatching of at least 90% of the embryos (control, 98.3 ± 1.67%; ammonium perchlorate at 1 mg/L, 96.7 ± 1.67%; 10 mg/L, 98.3 ± 1.67%; 100 mg/L, 93.3 ± 1.67%). Thereafter, percentage survival was unaffected by exposure to ammonium perchlorate (control, 79.7 ± 10.3%; ammonium perchlorate at 1 mg/L, 70.3 ± 7.15%; 10 mg/L, 74.7 ± 11.30%; 100 mg/L, 82.0 ± 9.40%).

### Development and growth.

**Figure 2:**
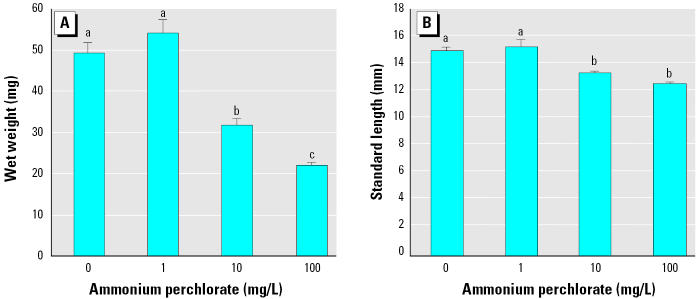
Wet weight (*A*) and standard length (*B*) of fathead minnows exposed to ammonium perchlorate from day 0 to day 28 (*n* = 30–36). Groups that are not significantly different are denoted by the same letter. Values shown are mean ± SEM.

After 28 days (posthatch day 23), fish exposed to 10 mg/L and 100 mg/L ammonium perchlorate were visibly smaller than fish exposed to 1 mg/L ammonium perchlorate or controls. Wet weight (*p* < 0.05; Figure 2A) and standard length (*p* < 0.05; Figure 2B) were significantly lower in fish exposed to 10 mg/L and 100 mg/L ammonium perchlorate compared with fish exposed to 1 mg/L perchlorate and controls.

Perchlorate exposure at the two higher concentrations resulted in delayed development. On study day 28, fish exposed to 10 and 100 mg/L had minimal appearance of scales, and the gut was still visible through the sides of the fish. In contrast, control fish had developed scales and pigmentation such that their viscera were no longer visible externally.

### Histology.

**Figure 3:**
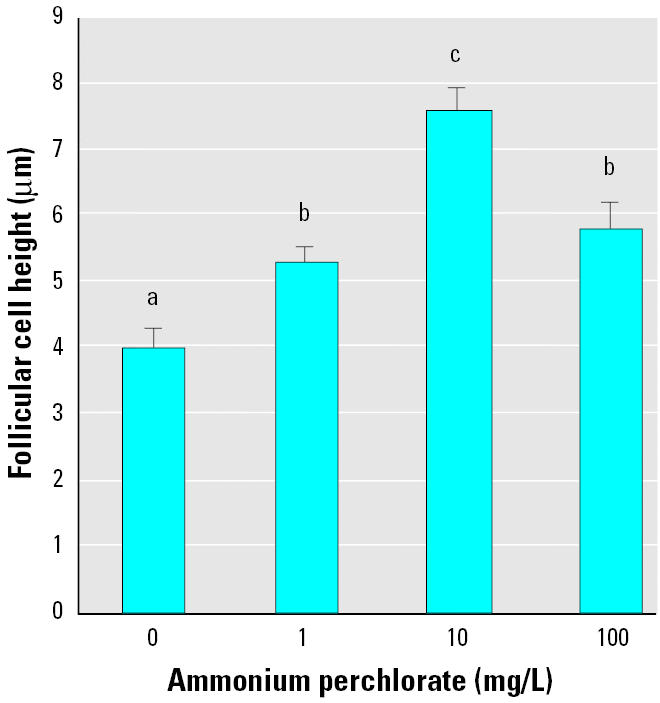
Follicular epithelial cell height of thyroid follicle of fathead minnows exposed to ammonium perchlorate from day 0 to day 28. These data are derived from five fish per treatment and between 5 and 13 follicles per fish. Groups that are not significantly different are denoted by the same letter. Values shown are mean ± SEM.

**Figure 4:**
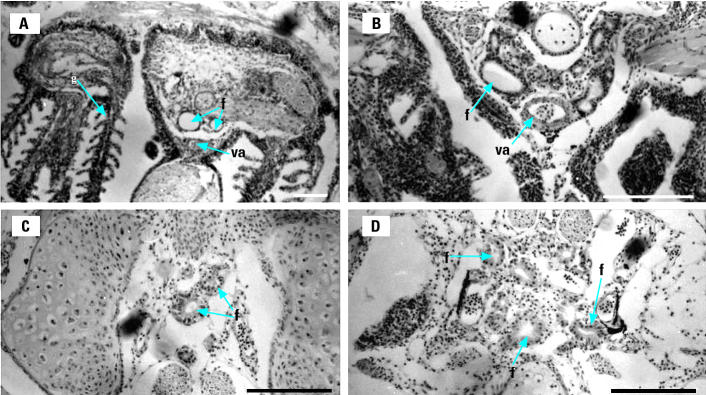
Thyroid follicles (f) in close proximity to the ventral aorta (va) and gills (g) of fathead minnows exposed to ammonium perchlorate from day 0 to day 28. (*A*) Control. (*B*) 1 mg/L ammonium perchlorate. (*C*) 10 mg/L ammonium perchlorate. (*D*) 100 mg/L ammonium perchlorate. Bars = 100 μm.

Fish exposed to all ammonium perchlorate concentrations exhibited significantly greater thyroid follicular epithelial cell height than did control fish (*p* < 0.05; Figure 3). Control fish had cuboidal follicular epithelial cells (Figure 4A), and the central colloid was full or showed only slight vacuolation. Individual fish exposed to 1 mg/L ammonium perchlorate showed a range of states of follicular colloid, from full colloid to visible vacuolation of the lightly stained central colloid (Figure 4B). In fish exposed to 10 mg/L, all follicles had more columnar epithelial cells (Figures 3 and 4C) and reduced follicular colloid (Figure 4C). Fish exposed to 100 mg/L ammonium perchlorate showed greatly enlarged epithelial cells (Figures 3 and 4D) and reduction or apparent absence of colloid (Figure 4D).

### Whole-body thyroid hormone content.

**Figure 5:**
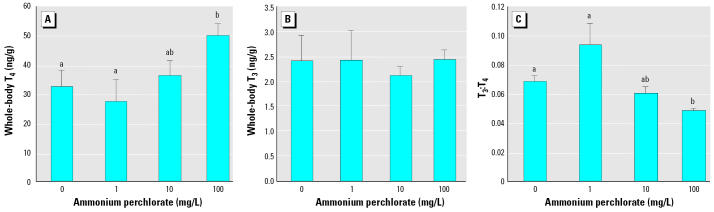
Whole-body T_4_ (*A*), T_3_ (*B*), and T_3_:T_4_ ratio (*C*) in fathead minnows exposed to ammonium perchlorate from embryo from day 0 to day 28 (*n* = 15–18). Groups

Whole-body T_4_ was significantly higher in fish exposed to 100 mg/L ammonium perchlorate than in either fish exposed to 1 mg/L or control fish (*p* < 0.05; Figure 5A). In contrast, there was no significant difference between the whole-body T_3_ content of con-trol fish and that of any group of fish exposed to ammonium perchlorate (Figure 5B). In line with these findings, thyroid hormone ratios (T_3_:T_4_) were significantly decreased in fish exposed to 100 mg/L perchlorate compared with control fish (*p* < 0.05; Figure 5C).

## Discussion

Exposure of fathead minnow embryos to ammonium perchlorate had no significant effect on their hatching, in agreement with a similar lack of effect of ammonium perchlorate on hatching by the amphibian *Xenopus laevis* at concentrations of < 1,000 mg/L (Goleman et al. 2002). Thereafter, however, exposure to ammonium perchlorate resulted in developmental retardation, both in *X. laevis*, where a reduced snout to vent length was observed after 16 days exposure of larvae to 425 mg/L ammonium perchlorate (Goleman et al. 2002), and in the present studies of fathead minnows exposed to 10 and 100 mg/L ammonium perchlorate.

The transition from larvae to juvenile fish in cyprinids such as the zebrafish and fathead minnow is characterized by the formation of scales, alongside other developmental changes (Brown 1997). In the present studies, development of scales and pigmentation was delayed in fathead minnows exposed to 10 mg/L and 100 mg/L ammonium perchlorate, indicating that the larval to juvenile transition in these fish had not been completed within the 28-day study period, whereas control fish successfully completed this transition. Impeded development and growth of fathead minnows held in perchlorate solutions was also indicated by the significantly lower wet weight and body length of fish exposed for 28 days to 10 mg/L and 100 mg/L ammonium perchlorate.

The reduced growth and inhibited development of fathead minnows exposed to the two higher concentrations of perchlorate is highly likely to reflect the cumulative impacts of ammonium perchlorate on thyroid status over the time course of the 28-day period of exposure, and further studies are now needed to define the effects of perchlorate on growth and development at earlier time points as well as their longer-term implications. Thyroid hormones are well known to play an important role in larval metamorphosis in flatfish (de Jesus et al. 1993; Inui et al. 1995) and in other species, such as the grouper, that undergo similar dramatic morphologic changes during metamorphosis from planktonic larvae to bottom dwellers (Trijuno et al. 2002), but the specific roles of thyroid hormones in regulating developmental processes in other teleostan fishes are less well defined. Our studies of fathead minnows have shown a peak in both T_4_ and T_3_ at 9 and 16 days posthatch, respectively (Crane 2003; Crane et al. 2004), suggesting roles in regulating particular developmental processes at this time, such as cartilage and gut formation (Liu and Chan 2002). Furthermore, thyroid hormones have been implicated in regulating the growth of both larval and adult fish, acting either directly or indirectly, via stimulation of growth hormone or insulin-like growth factors (Boeuf et al. 1989; Deane et al. 2003; Ebbesson et al. 1998; Marti-Palanca and Perez-Sanchez 1994; Perez-Sanchez and Le Bail 1999; Woo et al. 1991).

Pharmacologic inhibition of thyroidal uptake of iodide by perchlorate (Capen 1997; Wolff 1998) has been associated with reduced circulating levels of thyroid hormones in studies of mammals and birds given ammonium perchlorate in drinking water (McNabb et al. 2004; Siglin et al. 2000; York et al. 2001a, 2004). Similarly, low circulating concentrations of T_4_ and T_3_ have been reported in fish exposed to potassium perchlorate at 100–500 mg/L (Manzon and Youson 1997; Manzon et al. 1998). However, in our studies, despite the observed effects of ammonium perchlorate on growth and development of fathead minnows achieved over the 28-day experiment, there was no evidence of a significant depression in whole-body thyroid hormones after this period of exposure.

Thyroid function involves a series of sequential steps, beginning with the acquisition of iodide. Fish have a well-developed capacity to take up iodide from the environmental medium across their gills and, unlike mammals, do not have to rely on the dietary supply (Eales and Brown 1993). As a result, there is little evidence of natural thyroid deficiencies. Nevertheless, it is possible that the changing supply of dietary iodide in our studies may have had significance when set against the perchlorate inhibition of iodide uptake. In our studies, the developing fathead minnows were initially fed on rotifers and later weaned onto brine shrimp hatched in saline and a resultant increase in dietary iodide could have contributed to an elevated iodide:perchlorate ratio, aiding in the regulation of whole-body T_4_ despite perchlorate exposure.

A further major influence on circulating thyroid hormones during our study would have been the natural regulatory systems in fish that normally achieve stable circulating levels. In fish, circulating T_3_ is almost exclusively determined by peripheral deiodination (Eales and Brown 1993; Van Putte et al. 2001), whereas T_4_ levels are self-regulating via the pituitary–thyroid axis. T_4_ plays a major role is regulating pituitary release of thyroid-stimulating hormone (TSH), whereas in fish, in contrast to the situation in mammals, T_3_ has been found to exert no significant feedback regulation on TSH release (Eales and Brown 1993). Thus, the pituitary–thyroid axis responds to increases or decreases in plasma T_4_ concentrations, initiating compensatory changes in the thyroid activity and potential restoration of the T_4_ level. Therefore, the effects of perchlorate on the iodide uptake of thyroidal tissue in fathead minnows would be predicted to initially that are not significantly different are denoted by the same letter. Values shown are mean ± SEM. depress circulating levels of T_4_ resulting in stimulation of the pituitary release of TSH and activation of thyroid tissue to attempt to regulate thyroid hormone levels. Despite the apparent lack of a depression in either T_4_ or T_3_ content after 28 days of exposure to per-chlorate, our histologic results showed a marked hypertrophy of follicular epithelial cells. Therefore, the observed thyroid hyperplasia after 28 days of exposure to perchlorate is very likely to have resulted from a hypothyroidism that occurred during the 28-day study period and hence a reduced negative feedback on the pituitary, stimulating release of TSH. Similar thyroid hyperplasia has been observed after treatment of fish with goitrogens such as thiourea and thiouracil that inhibit coupling and formation of iodotyrosines in the thyroid. These studies have, furthermore, shown dose-dependent effects with maintenance of circulating T_4_ at low levels of goitrogen but depressed circulating T_4_, despite a more pronounced increase in epithelial cell height of thyroid tissue at higher doses (Eales and Brown 1993).

Thyroid gland hyperplasia during prolonged perchlorate exposure has also been reported in the amphibian *Bufo arenarum* exposed to potassium perchlorate at 340 mg/L for 5 months (Miranda et al. 1996). Our studies provide evidence of thyroid hyperplasia in fathead minnows at all concentrations of perchlorate investigated, and over a relatively short time frame. In fathead minnows, a 1-month period of exposure to as little as 1 mg/L initiated a significant increase in epithelial cell height. Exposure to 10 mg/L ammonium perchlorate increased the normally cuboidal epithelium with epithelial cell heights of 4 ± 0.27 μm to a columnar epithelium with cell height of 7.6 ± 0.35 μm. In contrast, *B. arenarum* exposed for 5 months to 340 mg/L potassium perchlorate showed a more pronounced stimulation of thyroid hypertrophy with epithelial cell height increased from 7 to 23 μm.

Despite the histologic evidence of hypothyroidism in all perchlorate-exposed fathead minnows, there was no evidence of a sustained depression in whole-body content of thyroid hormones, and by the end of the 28 days of exposure to 100 mg/L ammonium perchlorate, T_4_ was significantly elevated compared with that of the control fathead minnows.

The stimulation of the thyroid follicles by increased TSH (Eales and Brown 1993) may have subsequently compensated for the perchlorate inhibition of iodide uptake, restoring (1 or 10 mg/L ammonium perchlorate) or even elevating T_4_ (100 mg/L ammonium perchlorate). This regulation may have been aided by increased iodide gained from the diet of brine shrimp and a reduced perchlorate:iodide ratio. An increased release of stored thyroid hormone from the thyroid follicles (again stimulated by TSH) is also a possible contributory factor. Further studies using pronase digestions of thyroidal tissues could provide information of hormone stores bound to thyroglobulin (Kowalczyk and Sotowska-Brochocka 2000; Plohman et al. 2002) and the changes during perchlorate exposure. Our histologic studies showed a marked reduction in colloid within the follicles after 28 days of exposure to perchlorate, and in the longer term a more persistent depression in T_4_ may therefore occur.

Although our studies indicated elevated whole-body T_4_ content at the highest concentration of perchlorate (100 mg/L) after 28 days of exposure, T_3_, the hormone that exerts the principal physiologic effects (Eales and Brown 1993), was unchanged. As a result, T_3_:T_4_ ratios were significantly reduced. Regulation of plasma T_3_, despite depressed plasma T_4_, was reported in rabbits and rats given perchlorate in drinking water (York et al. 2001a), although time-related impacts of perchlorate in drinking water with longer- term depression of both circulating T_4_ and T_3_ may occur as regulation breaks down (Siglin et al. 2000). Our study of fathead minnows only extended for 28 days, and further studies over a longer time period are now warranted to gain a fuller picture of the potential impacts of this pervasive thyroid toxicant in the wild.

## Conclusions

The results reported here indicate that environmentally relevant levels of ammonium perchlorate are likely to affect the thyroid axis of teleost fish. Growth and development of the early life stages of fathead minnows were significantly retarded after a 28-day exposure to 10 or 100 mg/L perchlorate, and we suggest that these changes are the result of hypothyroidism during the early stages of exposure. Our histologic studies showed that thyroid follicular epithelial cell height is a sensitive and appropriate biomarker for perchlorate exposure in aquatic vertebrates. However, after 28 days of exposure to perchlorate, fathead minnows achieved homeostasis of the major physiologically active hormone T_3_ and T_4_ levels were similar in control fish and all perchlorate-exposed fish except at the highest concentration (100 mg/L). Further studies are needed to investigate whether there is up-regulation of T_4_ production or increased release at this stage in exposure. It also remains to be determined how fathead minnows exposed to perchlorate would survive and function in the longer term and, given the impeded growth and development, whether these fish would reach sexual maturity.
